# Large Language Model–Based Responses to Patients’ In-Basket Messages

**DOI:** 10.1001/jamanetworkopen.2024.22399

**Published:** 2024-07-16

**Authors:** William R. Small, Batia Wiesenfeld, Beatrix Brandfield-Harvey, Zoe Jonassen, Soumik Mandal, Elizabeth R. Stevens, Vincent J. Major, Erin Lostraglio, Adam Szerencsy, Simon Jones, Yindalon Aphinyanaphongs, Stephen B. Johnson, Oded Nov, Devin Mann

**Affiliations:** 1 NYU Grossman School of Medicine, New York, New York; 2 NYU Stern School of Business, New York, New York; 3 NYU Tandon School of Engineering, New York, New York

## Abstract

**Importance:**

Virtual patient-physician communications have increased since 2020 and negatively impacted primary care physician (PCP) well-being. Generative artificial intelligence (GenAI) drafts of patient messages could potentially reduce health care professional (HCP) workload and improve communication quality, but only if the drafts are considered useful.

**Objectives:**

To assess PCPs’ perceptions of GenAI drafts and to examine linguistic characteristics associated with equity and perceived empathy.

**Design, Setting, and Participants:**

This cross-sectional quality improvement study tested the hypothesis that PCPs’ ratings of GenAI drafts (created using the electronic health record [EHR] standard prompts) would be equivalent to HCP-generated responses on 3 dimensions. The study was conducted at NYU Langone Health using private patient-HCP communications at 3 internal medicine practices piloting GenAI.

**Exposures:**

Randomly assigned patient messages coupled with either an HCP message or the draft GenAI response.

**Main Outcomes and Measures:**

PCPs rated responses’ information content quality (eg, relevance), using a Likert scale, communication quality (eg, verbosity), using a Likert scale, and whether they would use the draft or start anew (usable vs unusable). Branching logic further probed for empathy, personalization, and professionalism of responses. Computational linguistics methods assessed content differences in HCP vs GenAI responses, focusing on equity and empathy.

**Results:**

A total of 16 PCPs (8 [50.0%] female) reviewed 344 messages (175 GenAI drafted; 169 HCP drafted). Both GenAI and HCP responses were rated favorably. GenAI responses were rated higher for communication style than HCP responses (mean [SD], 3.70 [1.15] vs 3.38 [1.20]; *P* = .01, *U* = 12 568.5) but were similar to HCPs on information content (mean [SD], 3.53 [1.26] vs 3.41 [1.27]; *P* = .37; *U* = 13 981.0) and usable draft proportion (mean [SD], 0.69 [0.48] vs 0.65 [0.47], *P* = .49, *t* = −0.6842). Usable GenAI responses were considered more empathetic than usable HCP responses (32 of 86 [37.2%] vs 13 of 79 [16.5%]; difference, 125.5%), possibly attributable to more subjective (mean [SD], 0.54 [0.16] vs 0.31 [0.23]; *P* < .001; difference, 74.2%) and positive (mean [SD] polarity, 0.21 [0.14] vs 0.13 [0.25]; *P* = .02; difference, 61.5%) language; they were also numerically longer (mean [SD] word count, 90.5 [32.0] vs 65.4 [62.6]; difference, 38.4%), but the difference was not statistically significant (*P* = .07) and more linguistically complex (mean [SD] score, 125.2 [47.8] vs 95.4 [58.8]; *P* = .002; difference, 31.2%).

**Conclusions:**

In this cross-sectional study of PCP perceptions of an EHR-integrated GenAI chatbot, GenAI was found to communicate information better and with more empathy than HCPs, highlighting its potential to enhance patient-HCP communication. However, GenAI drafts were less readable than HCPs’, a significant concern for patients with low health or English literacy.

## Key Points

**Question:**

Can generative artificial intelligence (GenAI) chatbots aid patient–health care professional (HCP) communication by creating high-quality draft responses to patient requests?

**Findings:**

In this cross-sectional study of 16 primary care physicians’ opinions on the quality of GenAI- and HCP-drafted responses to patient messages, GenAI responses were rated higher than HCPs’ for communication style and empathy. GenAI responses were longer, more linguistically complex, and less readable than HCP responses; they were also rated as more empathetic and contained more subjective and positive language.

**Meaning:**

In this study, primary care physicians perceived that GenAI chatbots produced responses to patient messages that were comparable in quality with those of HCPs, but due to GenAI responses’ use of complex language, these responses could cause problems for patients with lower health or English literacy.

## Teaser

This cross-sectional study evaluates how primary care physicians rate draft responses to patient inbox messages written by generative artificial intelligence compared with those written by health care professionals.

## Introduction

The surge in patient-health care professional (HCP) messaging due to COVID-19 has increased electronic health record (EHR) inbox management burden,^1,2,3,4,5,6^ particularly for primary care physicians (PCPs), contributing to burnout (especially among female and Hispanic/Latino physicians).^3,7,8,9,10,11^ Each additional message adds more than 2 minutes of EHR time, encompassing message drafting, information searching, order placing, and documentation.^1,5,10^ Proposed relief strategies include EHR window switching reduction (improving user interface design), upskilling support staff, and billing for messages,^1,7,10,11^ a practice now permitted by the Centers for Medicare & Medicaid Services, which evidence suggests reduces messaging burden.^12^ Using EHR-integrated generative artificial intelligence (GenAI) chatbots to automate drafting responses to patient messages could streamline workflows and thus alleviate burnout.

GenAI chatbots are large language models (LLMs)^13^ that synthesize massive text volumes, including medical literature, and have potential in many health care applications, including clinical note generation and medical text simplification.^14,15,16,17^ Substantial implementation challenges include processing needs, model biases, privacy concerns, and absent evaluation benchmarks.^18,19,20,21,22^ Addressing these challenges will enhance understanding of this technology’s benefits and limitations.^18^ Successful adoption depends on understanding HCPs’ and patients’ perceptions of GenAI outputs.^13,16,18,20,21,23,24^

Studies investigating output quality, evaluation methods, and benchmarking are burgeoning as institutions pilot GenAI for in-basket messaging.^14,16,18,20,22,25,26,27,28,29,30,31,32,33,34^ Studies of physicians’ perceptions of GenAI response efficacy on various dimensions often find them equivalent to HCP-drafted responses, especially in empathy.^14,25,26,27,28^ Despite advocacy for including private information in AI models^20^ and the institutional push for EHR implementation,^35^ few studies have assessed chatbots’ use of private patient information to answer their messages.^32,33,34^

Our study addresses this gap by using private patient-HCP message-response pairs to investigate PCPs’ perceptions of GenAI drafts and explore underlying linguistic factors associated with equity and perceived empathy. We hypothesize that GenAI draft quality, assessed by PCPs on information content quality, communication style, and usability, will be equivalent to HCP-generated responses.

## Methods

This blinded cross-sectional quality improvement study evaluates PCP perceptions of GenAI responses to patient messages compared with HCP-generated responses. Subgroup analyses evaluated whether response quality varied with HCP type (physicians and nonphysicians) and patient message classification (laboratory results, medication refill requests, paperwork, and general medical advice; determined by the EHR’s proprietary message classification LLM [Epic]). Computational linguistics analyses compare response content to elucidate potential equity concerns and why drafts were considered empathetic. As part of an operational pilot program to implement and curate GenAI in-basket drafts most acceptable for end-users, this study met NYU criteria for quality improvement work and did not undergo institutional review board review. All study procedures complied with institutional ethical standards and those set by the Declaration of Helsinki and are reported using the Strengthening the Reporting of Observational Studies in Epidemiology (STROBE) reporting guidelines for cross-sectional studies.

### Study Setting and Participants

A convenience sample of 16 PCPs were recruited from a large urban academic health system via a listserv email to 1189 internal medicine physician email addresses. PCPs affiliated with NYU Grossman School of Medicine were eligible. Participants provided consent by accepting the request to complete the survey and could opt out at any time. Surveys were collected between September 23 and November 16, 2023.

### Survey

Surveys were conducted in REDCap.^36^ Participants were provided a random selection of message-response pairs over 2 surveys, masked to whether the response was generated by GenAI or an HCP. The first contained 5 to 8 message-response pairs but no branching logic, while the second survey contained 15 to 20 message-response pairs and branching logic (eAppendix 1 in Supplement 1).

In both surveys, participants assessed the quality of response information content and communication style using 5-point Likert scale questions (scale 1-5, with 1 indicating strongly disagree and 5 indicating strongly agree), then answered whether it was preferable to starting from a blank page (usable vs unusable). Branching logic followed negative responses to Likert questions (bottom 2 box) to explore PCPs’ rationale, assessing for aspects like relevance and empathy. Regardless of whether a draft was considered usable, respondents selected from a list of items (eAppendix 1 in Supplement 1).

To construct the first survey, 200 random in-basket messages were extracted on September 12, 2023, including the corresponding HCP and AI-generated response. A total of 112 patient messages were reviewed, and 53 were excluded because they needed outside context (eg, laboratory values or medication names) for adequate evaluation of the response by participants, leaving 59 patient messages paired with both HCP and GenAI responses (52.7%). For the second survey, 500 random patient messages were extracted from the data warehouse on October 12, 2023, of which 464 were reviewed, and 146 were excluded due to the need for external context to properly evaluate the response, leaving 318 patient messages paired with both HCP and GenAI responses (68.5%) from which respondents’ questions were randomly assigned. Not all extracted messages were reviewed because the desired sample size (determined by the effort required of our participants) was achieved beforehand. Message-response pairs were randomly sampled (with replacement) for review, yielding some pairs being reviewed by different reviewers.

### Message-Response Sample Selection

The survey used in-basket message-response pairs from outpatient internal medicine departments participating in the pilot study of Generated Draft Replies (Epic), which generated responses using GPT-4 (OpenAI) through an EHR-integrated, vendor-prepared system. Pairs were randomly selected during the system’s silent validation, where drafts were being generated using Epic’s standard prompts but not seen by HCPs. The patient message subcategory (laboratory results, medication refill requests, paperwork, and general medical advice) determined which prompt (utilizing unique instructions and patient-specific details) generated the response (eg, laboratory results messages auto-populate recent test results, while medication refill requests include the active medication list). Evaluating standard prompts allows for benchmarking future prompt engineering efforts.

Inclusion criteria dictated that the first patient-initiated message between the patient and their HCP was chosen. If multiple HCP messages were sent in response, they were combined to minimize artificially incomplete responses. Responses from physicians, nurses, and frontline staff were included to reflect how patient requests are answered at many institutions.

### Statistical Analysis

Statistical analysis was conducted in Python version 3.9.16 (Python Software Foundation) in May 2024. We used a priori levels of significance of *P* < .05 for 2-sided tests of the null hypothesis that GenAI drafts would be equal to HCP responses on our 3 main survey questions. Mann-Whitney tests, robust to outliers and nonnormal distributions,^14,37,38^ evaluated differences between GenAI and HCP responses for the 2 main Likert questions and the 2-way paperwork messages subgroup comparison. Kruskal-Wallis tests compared the Likert scale means of physicians, nonphysicians, and GenAI across the 4 message subcategories.^14^ Independent sample *t* tests were used to compare differences in the proportion of GenAI vs HCP responses considered usable and all computational linguistics measures. One-way analysis of variance was used to compare the proportions of drafts considered usable by physicians, nonphysicians, and GenAI across 3 of 4 message subcategories. *P* values for all secondary analyses underwent a Sidak correction^39^ to account for multiple comparisons.

Because our data are ordinal and pairs were randomly assigned, the 1-way intraclass correlation coefficient (ICC) was used to estimate interrater reliability from the double-reviewed questions.^40^ Linear mixed models with random effects for individual reviewer variation and fixed effects for patient message subcategory and HCP subcategory were built (eAppendix 3 in Supplement 1) to assess how these factors affected survey results.

Computational linguistics methods analyzed responses’ length, complexity, and sentiment as well as the prevalence of specific content dimensions, such as positive emotion words. Such measures characterize writing styles and can anticipate readers’ attitudes and behavior toward the content, including their perception of its usefulness.^41^ Analysis was performed in Python with the pandas package (version 2.1.1) used to calculate word counts. Lexical diversity, or the variety of words used in a text, was assessed with the measure of textual, lexical diversity, calculated using the lexical_diversity package (version 0.1.1) and chosen due to its insensitivity to text length.^42,43,44,45^ Lexical diversity reflects language proficiency; highly diverse text indicates the author is using a broad range of vocabulary to express their thoughts and ideas.^46^ The textstat package (version 0.7.3) calculated the Flesch-Kincaid grade level, which is calculated from the average syllables per word and average words per sentence, and describes an English passage’s comprehensibility.^47,48^

Content analysis of the main response groups and empathetic subgroups utilized the latest Linguistic Inquiry and Word Count (LIWC) application, LIWC-22, the preferred application for automated text analysis in social science research.^49^ LIWC-22 matches response words to various dictionaries and subdictionaries that represent themes like positive and negative emotion and reports metrics as a percentage of words in the text that exist in a given theme’s dictionary.^50,51,52,53^ The textblob package (version 0.17.1) facilitated sentiment analysis, a common method used to assess subjectivity (range 0 to 1, higher indicating more subjectivity) and polarity, or the overall positive/negative tone of a text (range −1 to 1, higher is more positive).^54,55,56^

## Results

Of 1189 email addresses on the internal medicine listserv, 16 PCP participants (1.3%) volunteered. All were outpatient faculty, 8 (50.0%) were female, 7 (43.8%) worked primarily at NYU Langone Health, 5 (31.2%) at Bellevue Hospital, and 2 (12.5%) at the Manhattan Veteran’s Affairs hospital.

Of 344 evaluated survey message-response pairs (175 GenAI drafted; 169 HCP drafted), there were 157 single-reviewed, 73 double-reviewed, 11 triple-reviewed, and 2 quadruple-reviewed message-response pairs, resulting in 117 unique HCP and 126 unique GenAI message-response pairs. Branching logic only occurred during the second survey and was available for 207 unique questions (85.2%).

### Survey Results

**Figure 1.**
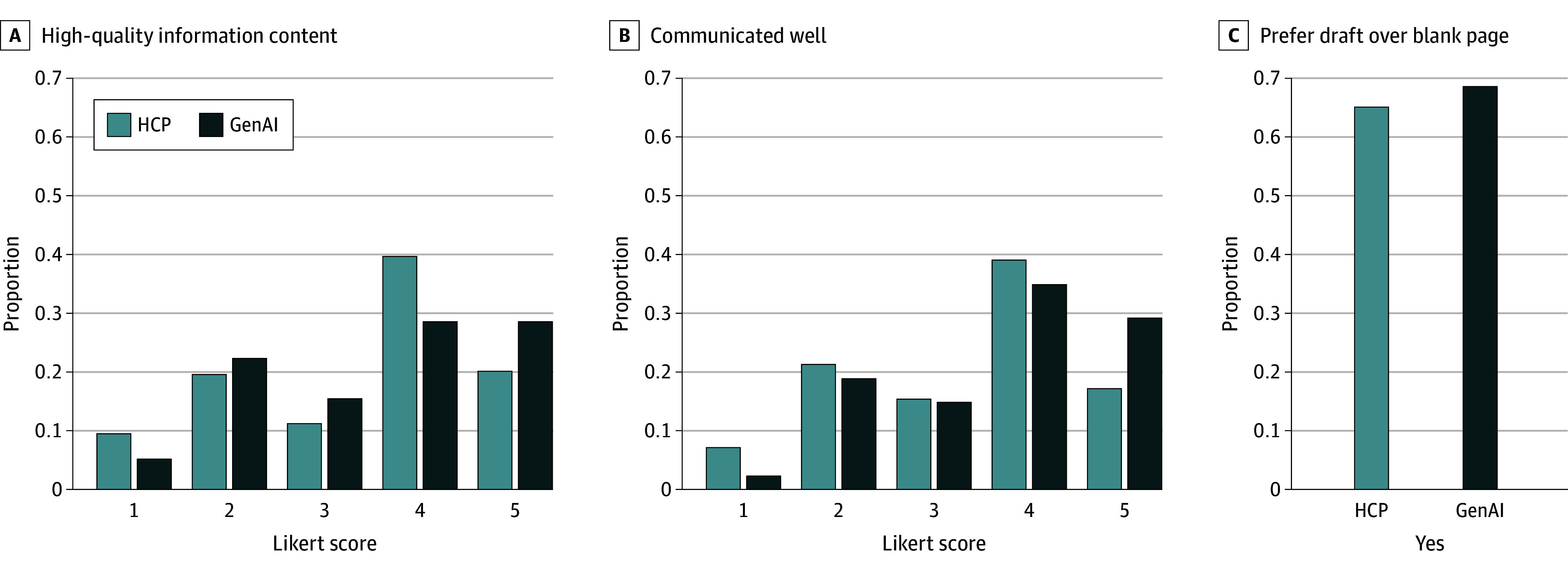
Distribution of Health Care Professional (HCP) and Generative Artificial Intelligence (GenAI) Responses to Each Main Survey Question

Participant evaluations were generally positive for GenAI and HCP responses (Figure 1 and Table 1). ICC (range −1 to 1) was 0.11 for information content quality, 0.094 for communication style, and 0.012 for draft usability. Low ICC results were not explained by message source (HCP vs GenAI) (eAppendix 5 in Supplement 1). To address ICC concerns, analyses treated multireviewed questions as independent observations rather than average their scores.

**Table 1.** Primary Care Physicians’ Ratings of HCP and GenAI Responses for Each of the Main Survey Questions

Message drafter	No.	Information content quality (5-point Likert), mean (SD)	*P* value	Communication quality (5-point Likert), mean (SD)	*P* value	Proportion of responses preferred to a blank page, mean (SD)	*P* value
HCP	169	3.41 (1.27)	.37	3.38 (1.20)	.01	0.65 (0.47)	.49
GenAI	175	3.53 (1.26)		3.70 (1.15)		0.69 (0.48)	

Abbreviations: GenAI, generative artificial intelligence; HCP, health care professional.

**Figure 2.**
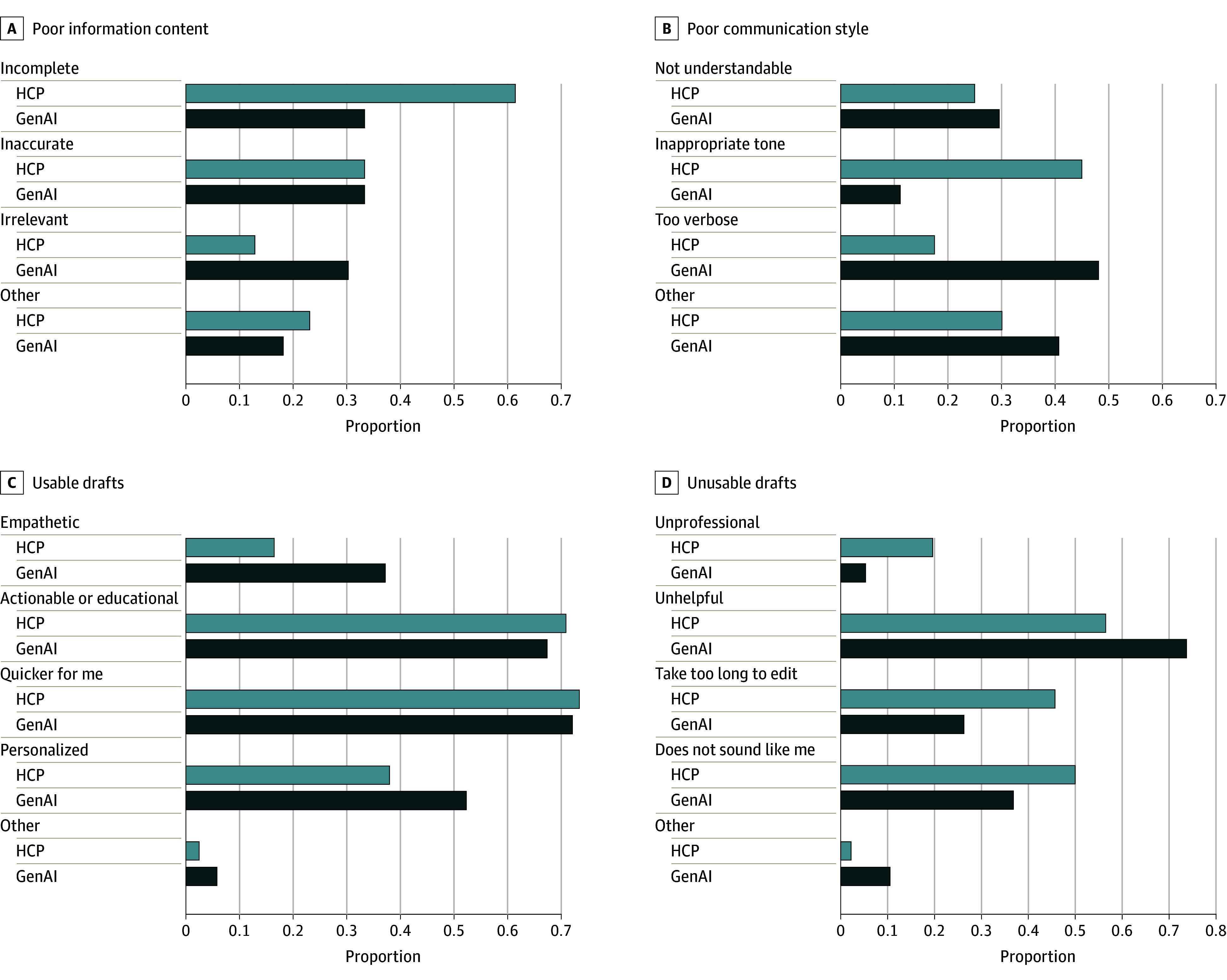
Branching Logic Results for Inadequate Information Content, Communication Style, and Both Usable and Unusable Draft Responses GenAI indicates generative artificial intelligence; HCP, health care professional.

The information content quality (accuracy, completeness, and relevance) of GenAI and HCP responses did not differ statistically (mean [SD], 3.53 [1.26] vs 3.41 [1.27]; *P* = .37; *U* = 13 981.0), a finding that persisted when controlling for individual reviewer variance, HCP subcategory (physician vs nonphysician) as a random effect, and patient message subcategory as a fixed effect (eAppendix 3 in the Supplement). For responses with inadequate information content quality (Likert score <3), HCP responses, compared with GenAI responses, were more often incomplete (24 of 39 [61.5%] vs 11 of 33 [33.3%]) while GenAI responses, compared with HCP responses, were more often irrelevant (10 [30.3%] vs 5 [12.8%]) (Figure 2). The other category for HCP responses (9 [23.1%]) received comments on unresponsiveness or even rudeness (eAppendix 4 in Supplement 1), while GenAI received comments (6 [18.2%]) about insensitivity to clinical urgency.

GenAI responses significantly outperformed HCP responses in communication style (understandability, tone, verbosity; mean [SD], 3.70 [1.15] vs 3.38 [1.20]; *P* = .01; *U* = 12 568.5). Subgroup analyses (eAppendix 2 in Supplement 1) and linear mixed models (eAppendix 3 in Supplement 1) revealed that physician underperformance was associated with this discrepancy. Low-scoring responses (Likert score <3) criticized HCPs more than GenAI for inappropriate tone (18 of 40 [45.0%] vs 3 of 27 [11.1%]) and criticized GenAI more than HCPs for verbosity (13 [48.1%] vs 7 [17.5%]). Free-text comments (HCP, 11 [30.0%]; GenAI, 12 [40.7%]) highlighted HCPs’ use of jargon and GenAI’s extraneous information.

GenAI and HCP responses were considered usable in similar proportions (mean [SD] proportion usable, 0.69 [0.48] vs 0.65 [0.47]; *P* = .49; *t* = −0.6842). Overall, 50% or less of the physician and laboratory results responses were considered usable (eAppendix 2 in Supplement 1). For unusable drafts, criticisms were evenly spread for HCPs, while GenAI was more often deemed unhelpful (28 of 38 [73.7%]). Insufficient professionalism occurred almost 4 times as frequently in the HCP responses vs GenAI responses (9 [19.6%] vs 2 [5.3%]). HCPs’ and GenAI’s free-text comments (1 [2.2%] vs 4 [10.5%]) both mentioned inadequate concern with a message’s clinical urgency, and GenAI responses were criticized for insufficient reasoning for proposed actions.

PCPs noted drafts were usable mainly because they would have been quicker to edit than start anew (58 of 79 HCP drafts [73.4%]; 62 of 86 GenAI drafts) and were more actionable or educational (56 HCP drafts [70.9%]; 58 GenAI drafts [67.4%]). GenAI responses, compared with HCP responses, were more often perceived as personalized (45 [52.3%] vs 30 [38.0%]) and empathetic (32 [37.2%] vs 13 [16.5%]; difference, 125.5%). Comments on HCP (2 [2.5%]) and GenAI (5 [5.8%]) responses critiqued clarity, and some GenAI responses were identified as computer-generated.

### Computational Linguistics Results

GenAI responses were 38% longer than HCPs’ (imposing a burden on readers’ time), but the difference was not statistically significant (mean [SD] word count, 90.5 [32.0] vs 65.4 [62.6]; *P* = .07; difference, 38.4%); had greater lexical diversity (requiring a wider vocabulary for readers to comprehend) (mean [SD] score, 125.2 [47.8] vs 95.4 [58.8]; *P* = .002; difference, 31.2%), and required higher levels of education to understand (mean [SD] Flesch-Kincaid grade level, 8.1 [1.6] vs 6.5 [2.3]; *P* < .001) (Table 2). GenAI responses utilized more polarity (positivity) (mean [SD], 0.21 [0.14] vs 0.13 [0.25]; *P* = .02; difference, 61.5%) and subjectivity (mean [SD], 0.54 [0.16] vs 0.31 [0.23]; *P* < .001; difference, 74.2%), a pattern maintained in empathetic responses, which also contained a significantly higher proportion of (particularly positive) emotion words and a greater use of affiliative language (Table 3).

**Table 2.** Basic Computational Linguistics and Lexical Complexity Metrics per Unique Responses in Each Group

Metric	Mean (SD)		*P* value
	HCP (n = 117)	GenAI (n = 126)	
Word count	65.4 (62.6)	90.5 (32.0)	.07
Measure of textual lexical diversity	95.4 (58.8)	125.2 (47.8)	.002
Flesch-Kincaid grade level	6.5 (3.3)	8.1 (1.6)	<.001

Abbreviations: GenAI, generative artificial intelligence; HCP, health care professional.

**Table 3.** Content and Sentiment Analysis of GenAI vs HCP and Empathetic vs Nonempathetic Responses

Metric	Mean (SD)		*P* value	Mean (SD)		*P* value
	HCP (n = 117)	GenAI (n = 126)		Empathetic (n = 41)	Nonempathetic (n = 166)	
Polarity	0.13 (0.24)	0.21 (0.14)	.02	0.26 (0.21)	0.15 (0.19)	.04
Subjectivity	0.31 (0.23)	0.54 (0.16)	<.001^2^	0.56 (0.18)	0.40 (0.22)	.003
Affiliation	1.92 (2.46)	2.51 (1.97)	.56	3.06 (2.01)	1.98 (2.20)	.08
Emotion	1.21 (2.28)	1.41 (1.42)	.99	2.42 (2.06)	1.12 (1.82)	.002
Positive emotion	0.87 (2.12)	1.20 (1.38)	.95	1.95 (2.14)	0.86 (1.67)	.01
Negative emotion	0.29 (1.05)	0.20 (0.55)	.99	0.41 (0.95)	0.22 (0.87)	.99

Abbreviations: GenAI, generative artificial intelligence; HCP, health care professional.

## Discussion

This analysis suggests that GenAI draft responses to patient requests, rated similarly to HCPs responses, could help mitigate the growing burden of in-basket messages, a known contributor to physician burnout.^1,2,3,4,57^ According to the PCP respondents, and consistent with prior studies,^25,26,27,28^ GenAI drafts outperformed HCPs’ responses on communication quality. Despite poor interrater reliability, the sensitivity analysis revealed consistent patterns of findings even after incorporating random effects for reviewers. Subsequently including fixed effects for HCP and patient message subcategories revealed that physicians were responsible for HCP responses underperforming GenAI on communication quality. This may be because physicians responded to more challenging messages than their nonphysician colleagues.^11^

GenAI responses matched HCP responses in information quality, indicating effective use of health care–related training data^20^ and patient health data within the standard prompts. This deviates from Ayers et al,^26^ where chatbots had 3.6 times higher quality responses to public patient messages, but still supports chatbots’ utility. A crucial caveat is that intentional guardrails restrict the LLM’s confidence in providing medical information^22^ and are designed to limit hallucinations and automation bias,^14,18^ but may explain why PCPs found GenAI responses more often unhelpful and irrelevant.

GenAI’s poor performance on certain subgroups, especially laboratory results, likely results from the differing prompts by message type, reinforcing the need for benchmarking and thoughtful prompt engineering.^18,19,20^ Future implementers of GenAI into EHR in-basket messaging should direct resources toward revising prompts related to laboratory results.

The prevalence of affiliation words, positivity, and subjectivity in GenAI drafts may explain why they were perceived as more empathetic than HCPs’. Affiliation content, such as “together” and “us,” implies a partnership between the HCP and patient. Although empathy is context-sensitive, responses that PCPs perceived as empathetic contained more positive language, which may convey hopefulness and potentially better outcomes.^58^ GenAI could thus improve virtual communications between HCPs (physicians in particular) and patients. HCPs surprisingly did not leverage knowledge of their patients to communicate more empathetically than GenAI. GenAI’s more consistent language structure, reflected by smaller SDs for most metrics, and its use of more emotional and affiliative language, suggests PCPs may utilize structured responses that fill a gap in their typical responses.

Despite critiques of GenAI’s verbosity and readability, it maintained more appropriate tone and professionalism, potentially reflecting HCPs’ time constraints when drafting responses.^1,2,3,4,5,6,7,8,9,10,11^ In fact, PCPs cited quicker for me as the main reason drafts were considered usable. Although not the primary audience, PCPs must still perceive GenAI drafts as high quality before utilizing them. Patients are the ultimate recipients of drafts, and future research must assess their perceptions of GenAI responses, whose linguistic complexity may be preferred (or ignored) by physicians but burden those with low health or English literacy. Research must also explore concerns about whether GenAI perpetuates bias and health inequity of various patient demographic characteristics^32,33,34,59,60^ and determine whether communication gains outweigh such risks.

This study addressed GenAI implementation challenges, including benchmarking draft and prompt quality and understanding PCPs’ perceptions. A critical finding of our study was the inability of PCPs to agree with each other on what makes a draft high quality, suggesting that successful utilization of drafts by PCPs requires a personalized approach. Future research should investigate the impact of prompt refinement and personalization on end-users’ perceptions of draft quality. Computational linguistics may drive more intelligent prompt engineering to enhance outputs’ empathy, reduce their linguistic complexity, and improve personalization.

### Limitations

This study has limitations. Generalizability may be limited due to this study’s single-center focus and small sample size. The evaluated GenAI responses were not used to deliver patient care, which may limit our findings’ practical applicability. Low ICC suggests a need to adjust the survey questions or instructions (although variance in reviewer responses did not affect our findings) or conduct follow-up interviews to investigate reasons for disagreement. This study did not evaluate the perceptions of patients and nonphysician HCPs who participate in outpatient messaging. We acknowledge that for some HCPs who answer patient messages, particularly nonphysicians, templates are used to draft responses rather than a blank page; the presence of templates was not assessed, and future studies should treat templated HCP responses as a separate group for comparison. Furthermore, our study did not examine whether response quality varied with patient demographics.

## Conclusions

In this study, PCPs’ found EHR-integrated GenAI responses to private patient messages similar to HCPs in terms of information content quality, better with respect to communication style, and similar in their usability compared with starting from scratch. While poorly rated GenAI responses lacked relevance, were less helpful, or more verbose, they outperformed HCP responses in completeness, empathy, and professionalism. GenAI drafts acceptable to HCPs may offset the increasing workload (and diminishing well-being) they face from in-basket messages from patients. Future research should focus on optimizing the perceived quality of GenAI responses to end-users’, particularly patients’, perceptions; quantifying efficiency gains; and mitigating biases and hallucinations.

## Notes

Supplement 1. **eAppendix 1.** Survey Design Flow Diagram **eAppendix 2.** Subgroup Analyses Stratified First by HCP Type (Physician vs Nonphysician), Then by Patient Message Subcategory **eAppendix 3.** Linear Mixed Models With Random and Fixed Effects to Explore the Extent to Which Reviewer Variance or That Attributed to Patient Message (General Medical Advice, Laboratory Results, Medication Refill Requests, Paperwork) and HCP (Physician, Nonphysician) Subgroups Affected Final Results for the Main 3 Survey Questions **eAppendix 4.** Select Free-Text Comments From Each Questions When Other Was Chosen in the Branching Logic **eAppendix 5.** Exploration of Intraclass Correlation by Subgroup

Supplement 2.
Data Sharing Statement
