# Supplementary material for: Large Language Model–Based Responses to Patients’ In-Basket Messages
Source: JAMA Netw Open. 2024 Jul 16;7(7):e2422399. doi: 10.1001/jamanetworkopen.2024.22399 (PMC11252893; doi:10.1001/jamanetworkopen.2024.22399)
Supplement: Supplement 1. — eAppendix 1. Survey Design Flow Diagram eAppendix 2. Subgroup Analyses Stratified First by HCP Type (Physician vs Nonphysician), Then by Patient Message Subcategory eAppendix 3. Linear Mixed Models With Random and Fixed Effects to Explore the Extent to Which Reviewer Variance or That Attributed to Patient Message (General Medical Advice, Laboratory Results, Medication Refill Requests, Paperwork) and HCP (Physician, Nonphysician) Subgroups Affected Final Results for the Main 3 Survey Questions eAppendix 4. Select Free-Text Comments From Each Questions When Other Was Chosen in the Branching Logic eAppendix 5. Exploration of Intraclass Correlation by Subgroup [file jamanetwopen-e2422399-s001.pdf]

Supplemental Online Content

Small WR, Wiesenfeld B, Brandfield-Harvey B, et al. Large language model–based responses to patients' in-basket messages. *JAMA Netw Open.* 2024;7(7):e2422399. doi:10.1001/jamanetworkopen.2024.22399

eAppendix 1. Survey Design Flow Diagram

eAppendix 2. Subgroup Analyses Stratified First by HCP Type (Physician vs Nonphysician), Then by Patient Message Subcategory

eAppendix 3. Linear Mixed Models With Random and Fixed Effects to Explore the Extent to Which Reviewer Variance or That Attributed to Patient Message (General Medical Advice, Laboratory Results, Medication Refill Requests, Paperwork) and HCP (Physician, Nonphysician) Subgroups Affected Final Results for the Main 3 Survey Questions

eAppendix 4. Select Free-Text Comments From Each Questions When Other Was Chosen in the Branching Logic

eAppendix 5. Exploration of Intraclass Correlation by Subgroup

This supplemental material has been provided by the authors to give readers additional information about their work.

© 2024 Small WR et al. *JAMA Network Open.*

eAppendix 1. Survey Design Flow Diagram

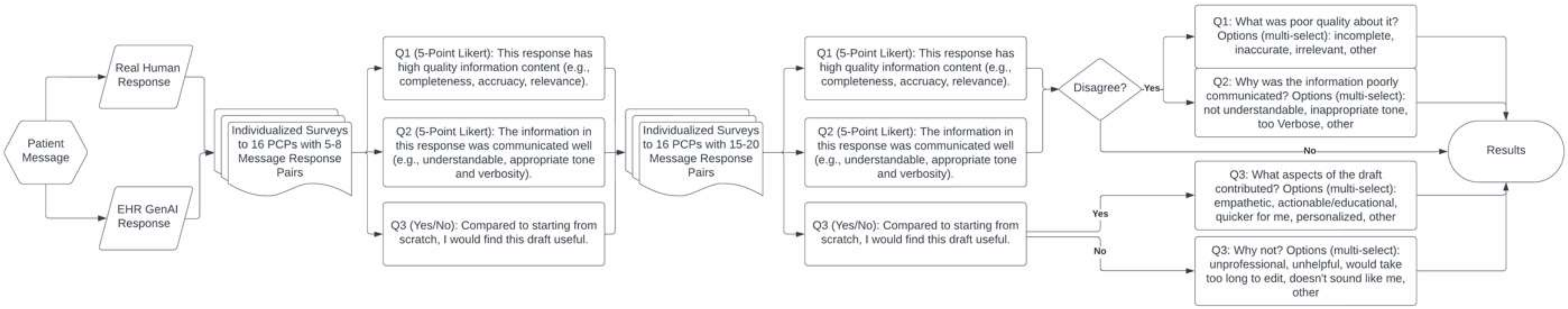

© 2024 Small WR et al. *JAMA Network Open.*

eAppendix 2. Subgroup Analyses Stratified First by HCP Type (Physician vs Nonphysician), Then by Patient Message Subcategory

Superscript indicates group similarity such that X[a] and Y[a] are similar while X[a] and Y[b] are statistically different.

Subgroup Analysis 1: Physicians vs Non-Physicians vs GenAI

Responder Type	n	Mean (SD) Information Content Quality (5-point Likert)	p-value	Mean (SD) Communication Quality (5-point Likert)	p-value	Proportion (SD) of Responses Preferred to a Blank Page	p-value
Physicians	38	3.37[a] (1.34)	0.96	3.00[a] (1.21)	0.012	0.50[a] (0.51)	0.18
Non-Physicians	131	3.43[a] (1.25)		3.49[b] (1.18)		0.69[a] (0.46)	
GenAI	175	3.53[a] (1.26)		3.70[b] (1.15)		0.69[a] (0.47)	

© 2024 Small WR et al. *JAMA Network Open.*

Subgroup Analysis 2: Physicians vs Non-Physicians vs GenAI Stratified by Patient Message Category

Patient Message Type	Responder Type	n	Mean (SD) Information Content Quality (5-point Likert)	p-value	Mean (SD) Communication Quality (5-point Likert)	p-value	Proportion (SD) of Responses Preferred to a Blank Page	p-value
General Medical Advice	Physicians	25	3.24[a] (1.36)	0.90	3.08[a] (1.22)	0.0068	0.52[a] (0.51)	0.23
	Non-Physicians	60	3.53[a] (1.21)		3.43[b] (1.24)		0.75[a] (0.44)	
	GenAI	103	3.72[a] (1.18)		4.05[b] (0.93)		0.78[a] (0.42)	
Medication Refill Request	Physicians	5	3.40[a] (1.82)	0.99	2.20[a] (1.64)	0.90	0.20[a] (0.45)	0.91
	Non-Physicians	28	3.43[a] (1.35)		3.57[a] (1.14)		0.68[a] (0.48)	
	GenAI	30	3.83[a] (1.15)		3.40[a] (1.40)		0.67[a] (0.48)	
Lab Results	Physicians	8	3.75[a] (1.04)	0.33	3.25[a] (0.71)	0.36	0.62[a] (0.52)	0.76
	Non-Physicians	35	3.29[a] (1.25)		3.49[a] (1.15)		0.63[a] (0.49)	
	GenAI	37	2.70[a] (1.20)		2.86[a] (1.03)		0.43[a] (0.50)	
Paperwork	Physicians	0	N/A	0.99	N/A	0.98	N/A	0.99
	Non-Physicians	8	3.25[a] (1.39)		3.62[a] (1.19)		0.62[a] (0.52)	
	GenAI	5	4.00[a] (1.73)		4.40[a] (0.55)		0.80[a] (0.45)	

© 2024 Small WR et al. *JAMA Network Open.*

eAppendix 3. Linear Mixed Models With Random and Fixed Effects to Explore the Extent to Which Reviewer Variance or That Attributed to Patient Message (General Medical Advice, Laboratory Results, Medication Refill Requests, Paperwork) and HCP (Physician, Nonphysician) Subgroups Affected Final Results for the Main 3 Survey Questions

1. Linear mixed model including individual reviewers as random effects

Information Content				
	Coefficient (CI)	Std. Error	Z	P value
HCP	Reference	Reference	Reference	-
GenAI	0.16 (-0.10, 0.41)	0.13	1.2	0.23
Communication Style				
HCP	Reference	Reference	Reference	-
GenAI	0.35 (0.13, 0.57)	0.11	3.1	0.0020
Prefer to Blank Page (0 = No, 1 = Yes)				
HCP	Reference	Reference	Reference	-
GenAI	0.057	0.027	1.2	0.24

© 2024 Small WR et al. *JAMA Network Open.*

2. Linear mixed model including individual reviewers as random effects and patient message subcategory as fixed effects

Information Content				
	Coefficient (CI)	Std. Error	Z	P value
HCP	Reference	Reference	Reference	-
GenAI	0.14 (-0.11, 0.39)	0.13	1.1	0.28
General Medical Advice	Reference	Reference	Reference	-
Lab Results	-0.50 (-0.80,-0.19)	0.16	-3.2	0.0020
Medication Refill Requests	-0.010 (-0.34,0.33)	0.17	-0.038	0.97
Paperwork	-0.10 (-0.76,0.56)	0.34	-0.30	0.76
Communication Style				
HCP	Reference	Reference	Reference	-
GenAI	0.33 (0.11, 0.55)	0.11	3.0	0.0030
General Medical Advice	Reference	Reference	Reference	-
Lab Results	-0.40 (-0.67,-0.13)	0.14	-2.9	0.0040
Medication Refill Requests	-0.36 (-0.66,-0.064)	0.15	-2.4	0.017
Paperwork	0.25 (-0.33,0.83)	0.30	0.84	0.40
Prefer to Blank Page (0 = No, 1 = Yes)				
HCP	Reference	Reference	Reference	-
GenAI	0.047	0.048	1.0	0.33
General Medical Advice	Reference	Reference	Reference	-
Lab Results	-0.19 (-0.30,-0.070)	0.060	-3.1	0.0020
Medication Refill Requests	-0.11 (-0.24,0.019)	0.065	-1.7	0.094
Paperwork	-0.052 (-0.30,0.20)	0.13	-0.41	0.68

© 2024 Small WR et al. *JAMA Network Open.*

3. Linear mixed model including individual reviewers as random effects and both patient message and healthcare provider sub-categories as fixed effects

Information Content				
	Coefficient (CI)	Std. Error	Z	P value
GenAI	Reference	Reference	Reference	-
Physician	-0.23 (-0.64,0.19)	0.21	-1.1	0.28
Non-Physician	-0.11 (-0.38,0.16)	0.14	-0.80	0.42
General Medical Advice	Reference	Reference	Reference	-
Lab Results	-0.50 (-0.81,-0.19)	0.16	-3.2	0.0010
Medication Refill Requests	-0.015 (-0.36,0.32)	0.17	-0.089	0.93
Paperwork	-0.12 (-0.79,0.54)	0.34	-0.36	0.72
Communication Style				
GenAI	Reference	Reference	Reference	-
Physician	-0.81 (-1.2,-0.45)	0.18	-4.4	0.000
Non-Physician	-0.19 (-0.42,0.04)	0.12	-1.6	0.11
General Medical Advice	Reference	Reference	Reference	-
Lab Results	-0.43 (-0.70,-0.16)	0.14	-3.2	0.0020
Medication Refill Requests	-0.41 (-0.70,-0.11)	0.15	-2.7	0.0060
Paperwork	0.14 (-0.43,0.72)	0.29	0.49	0.62
Prefer to Blank Page (0 = No, 1 = Yes)				
GenAI	Reference	Reference	Reference	-
Physician	-0.22 (-0.38, -0.064)	0.052	-2.8	0.0060
Non-Physician	0.004 (-0.097,0.11)	0.052	0.082	0.935
General Medical Advice	Reference	Reference	Reference	-
Lab Results	-0.20 (-0.31,-0.083)	0.060	-3.1	0.0020
Medication Refill Requests	-0.13 (-0.25,0.0020)	0.065	-1.7	0.094
Paperwork	-0.090 (-0.34,0.16)	0.13	-0.71	0.48

© 2024 Small WR et al. *JAMA Network Open.*

eAppendix 4: Select Free-Text Comments From Each Questions When Other Was Chosen in the Branching Logic

	Inadequate Information Content	Inadequate Communication Style	Unusable Drafts	Usable Drafts
HCP	"Did not address the patient's concern.", 'Not helpful and robotic', 'Rude and non-responsive.'	'Patient needs a little more feedback, personalization', 'What does watch your diet mean- need to provide link to dietary practices', "Doesn't address patiet's worry and not clear they needed refills sent."	"It doesn't reflect my practice. ", 'The message should be responded to by the MD rather than telling the patient that the MD will eventually respond. ', 'I would worry about this patient'	'Would list which imaging study was ordered for improved clarity.', 'Concrete and clear follow up recommendations[JZ6] ', "I thought the person who responded did so politely, but most PCPs probably don't need to see the patient provide a jury duty letter and I don't like bringing my patients in only for that."
GenAI	"The patient has raised a concern that merits follow up sooner. It's not appropriate to put that on the patient at this juncture.", 'Response not relevant to question, non-human response', 'These results should be followed up by the person who ordered the test', 'It restates what she already knows that the scripts are wrong in pharmacy. '	'Included information about results but the patient did not ask for this information!', "Had the correct information- needs to be seen/evaluated but message was diluted by including information about 'current medication'.", 'It's not clear to me why the provider can't send it. If it's the front desk's responsibility, say that. "Added info that wasn't asked in the question about recent lab tests. "	'I would not want to provide medical advice without seeing the patient/talking to the patient', 'I would feel the need to start from scratch. I am worried about the patient, and the answer seems unrelated to my concern, which is her appropriate diagnosis and treatment. ', 'Not the right message. The patient should be told that the context of the illness must be understood before deciding about testing.',	"Clear a computer wrote this bc patient asking for an appointment and reply doesn't acknowledge that was the original request ", 'The message was sent to the provider but it appears that a team member responded; might make that more clear upfront in the message (e.g. I am so-and-so and work with Dr. X...)', 'Get the patient in to discuss this in person',

© 2024 Small WR et al. *JAMA Network Open.*

eAppendix 5. Exploration of Intraclass Correlation by Subgroup

	Information Content Quality	Communication Style	Draft Usability
ICC - Total	0.11	0.094	0.012
ICC - HCP Responses Only	0.236	-0.017	-0.07
ICC - GenAI Responses Only	-0.024	0.12	0.12

© 2024 Small WR et al. *JAMA Network Open.*
