# Supplementary material for: Large Language Model–Based Responses to Patients’ In-Basket Messages
Source: JAMA Netw Open. 2024 Jul 16;7(7):e2422399. doi: 10.1001/jamanetworkopen.2024.22399 (PMC11252893; doi:10.1001/jamanetworkopen.2024.22399)
Supplement: Supplement 2. — Data Sharing Statement [file jamanetwopen-e2422399-s002.pdf]

Data Sharing Statement

Small. Large Language Model–Based Responses to Patients' In Basket Messages. *JAMA Netw Open*. Published July 16, 2024. doi:10.1001/jamanetworkopen.2024.22399

Data
Data available: No
